# The Value of Making a Rectal Examination

**Published:** 1908-12-12

**Authors:** 


### Surgery.

### THE VALUE OF MAKING A RECTAL EXAMINATION.

The object of this article is to point out not only how much valuable information may be learned from examination of the rectum in a suitable case, but also the importance of always making such an examination in any case in which the symptoms are at all referable to a pelvic origin.

It is a regrettable fact, but none the less a true one, that this method of examination is often wilfully omitted. The writer had an opportunity some years back of examining the cases sent up weekly from all parts of the country to a special hospital for joint-diseases. Quite an appreciable proportion of the cases sent up with a diagnosis of sciatica or osteo-arthritis of the hip proved, on systematic examination, to be suffering from carcinoma of the rectum. A digital examination of the rectum would have saved the medical man from such an error of diagnosis in nearly every case; and the corollary is that any patient between the ages of forty-five and sixty complaining of pain referred to the hip-joint or to the sciatic nerve, especially on the left side, should always be examined by the rectum.

### In Disease of the Rectum.

It goes without saying that a rectal examination must always be made in a patient who complains of symptoms directly referable to the rectum itself. The information thus obtained is of enormous value. The presence of a carcinoma which is within reach of the examining finger—that is to say, within four inches from the anus—is immediately revealed; and not only its presence, but also its nature, its extent, and, last, but not least, the question whether it has infiltrated and become adherent to adjacent viscera. A small practical point of some value may be mentioned here, and that is that a growth may be just out of reach of the finger when the patient is examined lying down in the left lateral position, but if he is examined while standing up and then strains slightly the growth will come down so as to be palpable. The importance of being in possession of all this information about a rectal growth cannot be over-estimated, since in an early case one relies on these points in making up one's mind as to whether or not an extirpation of the rectum is justifiable. If, for instance, the growth can be plainly felt to be adherent to the sacrum behind (and it is then that symptoms simulating sciatica occur), or to the prostate in front, the answer must be in the negative, and the case must be watched, so that a colotomy can be performed at the proper moment, and that is as soon as symptoms of obstruction have made their appearance.

Any case of hæmorrhage from the rectum must also be examined digitally. The common causes are, of course, piles, carcinoma, and rectal polypus. The latter is a condition that is frequently missed. A rectal polypus is generally a pedunculated tumour, but its pedicle is often so much elongated as to form a stalk; and it is therefore enabled to slide out of the way of the examining finger; but this should not happen if it be borne in mind that the base of the stalk is usually attached to the anterior rectal wall.

In all cases where piles are complained of, or where the history points to this condition, a rectal examination must always be made. It must be remembered that the presence of piles is in some cases only a symptom of a graver trouble—namely carcinoma of the rectum. When this is the case, the patient is generally elderly, and the piles are associated with a relaxation of the sphincter ani and ballooning of the rectum. In ordinary cases of piles the condition found varies from that of merely a slight hæmorrhoidal congestion up to one in which the hæmorrhoids are definitely formed. In the former case palliative treatment will be sufficient, but in the latter nothing short of operation will rid the patient of his trouble. It is only by making a systematic examination of the rectum in all these cases that sufficient clinical experience can be accumulated to enable one to say definitely with which class of case one is dealing.

Then, again, in cases of fistula in ano, an examination should be made to find out the exact position of the internal opening. This will materially affect the prognosis with regard to the length of convalescence. It is a much more serious matter, for instance, if the internal opening is above the internal sphincter, so that both sphincters must be cut at the operation, than if it lies between the two, in which case only the external one need be divided.

### In Other Conditions.

So far we have dealt only with those cases in which the disease lies in the rectum itself, cases in which there is no excuse for omitting this manipulation; but much may also be learnt in conditions in which the rectum is not itself diseased, but is pressed upon or is secondarily involved. In this category may be classed certain cases in which an abscess has developed in connection with a dependent appendix. A bulging of the rectum will be felt upon the right side, which will certainly be tender, and often hot. Similarly, much information may be obtained as to the condition of the vesiculæ seminales and prostate, as might be expected from their intimate relation to the anterior wall of the rectum; in fact, only in this way can it be discovered if they are involved in a tuberculous infection which began in the testis or epididymis.

In a virgin suffering from pelvic trouble rectal examination must be relied on instead of vaginal; and a little practice enables one to obtain sufficiently accurate information in this way as to the position and size of the uterus and as to the presence of any abnormal swelling in connection with the pelvic organs.

Last, but not least, whenever tuberculous disease affecting the lower part of the vertebral column—*e.g.* lower lumbar vertebræ or sacrum, is suspected, a rectal examination should be made. The symptoms in these cases are often exceedingly vague, while all the time there may be a large pelvic abscess filling the pelvis and pressing on the rectum. The discovery of this will make the diagnosis clear.

